# Evolvable design of network-oriented services based on a core/periphery structure

**DOI:** 10.1038/s41598-023-38695-5

**Published:** 2023-07-19

**Authors:** Shiori Takagi, Shin’ichi Arakawa, Masayuki Murata

**Affiliations:** Graduate School of Information Science and Technology, Osaka University, 1-5 Yamadaoka, Suita, Osaka Japan

**Keywords:** Information technology, Computer science

## Abstract

In recent years, many new network-oriented services have emerged, and such services will need to be virtualized in the multi-access edge computing environment, which is currently being standardized along with fifth-generation network technology. The environment surrounding the service functions network changes over time, such as breaking changes of APIs, and these changes impact the services. The service design should be adaptable to user requirements and environmental changes for accommodating a large number of services at low cost. In addition, it is required not only to assume environmental changes when initially designing the service functions network, but also to enable the network to continue to change its structure to adapt to new environmental changes in the future. In this paper, we propose a method to evolve the entire network of service functions based on a core/periphery structure. The advantage of the core/periphery structure is that it helps reduce the costs for maintaining or changing services by dividing the service functions into core and periphery functions. We propose a method to evolve a service functions network based on this core/periphery structure. Our method evolves the structure of the service functions network at low cost by keeping the core and peripheral functions at the appropriate scale. In addition, our proposed method accommodates almost 100% of randomly generated service chains, and holds their length to less than twice the minimum chain length. Our simulation results reveal that the structure of the service functions networks can continue to evolve at a low cost and maintain a high service accommodation ratio.

## Introduction

In recent years, many new network-oriented services have emerged, and information networks have been changing rapidly. For example, rapidly developing telepresence^1^ services allow human users to experience being present in a remote place virtually by operating a remote robot as if it is their own body and using other virtual reality or mixed reality technologies. Several different services that are yet to be realized are expected to emerge in the future. Such services will need to be virtualized in the multiaccess edge computing environment^2–4^, which is currently being standardized along with fifth-generation network technology.

Network-oriented services consist of service functions deployed on multiple devices. The service functions have been developed by various developers, and some of them are available from other service functions via application interfaces (APIs). That is, a network of service functions is formed and made available. Some of the service functions are combined as a service chain by the service provider and provided to meet user requirements.

The environment surrounding the network changes over time. For example, as requirements for new services increase, new service functions may be developed, or the popularity of some service functions may change. In addition, due to breaking changes of APIs, users may not be able to use the service, and service providers may have to create new service chains. Xavier et al.^5^ examined API changes and their impact on Java libraries and client applications, and found that the number of APIs affected increases over time; the frequency of breaking changes to a given API increases over time, from about 0.29 times in the first year of development to about 0.49 times in the fifth year. Against the breaking change or other possible changes, some of services are reconfigured to use the new or alternative APIs. Reconfiguration of the services and their accommodation requires development costs, including the development cost of interfaces for connection to new or alternative APIs. Thus, development costs will become high when new interfaces are developed each time they are required or when they are connected to many APIs in advance. Therefore, an adaptable service functions network design is required to allow the service functions network to accommodate new services at low cost. Adapting to the environmental changes requires the addition of interfaces between service functions or the development of new service functions. Thus, when design decisions that are expedient in the short term are made, the costs of maintaining and adapting this system in the future increase, which is known as technical debt^6^.

By predicting the API changes and services that are likely to require to be accommodated newly and connecting the service functions required for them with fewer interfaces, an adaptable service functions network can be composed at that time. However, it cannot meet new unpredictable service demands. It is difficult to predict changes in a service functions network consisting of service functions developed by different developers. Therefore, a service functions network that continue to adapting to unpredictable changes with low development costs is required.

We refer to the service functions network that changes the structure at low cost with maintaining the ability to provide new services, as an evolvable service functions network.

Algorithms with evolvability include genetic algorithms (GAs) and evolution strategies, which have been applied to a variety of problems^7,8^. However, these algorithms are based on the presumption that the problem to be solved is described as an optimization problem. Environmental changes in service functions networks include a significant increase of service functions, and new, seldom used services can grow, for example, due to increased popularity, but it is difficult to predict what environmental changes will occur in the future. The environmental changes that may occur in the future are too numerous to list, and it is difficult to mathematically represent future environmental changes as goals of optimization problems. Thus, methods of dealing with traditional optimization problems cannot maintain a service functions network evolvable in the long term.

We have been investigating a core/periphery structure^9,10^ that allows service components to effectively adapt to each user request and environmental variation. The core/periphery structure is a model for flexible and efficient information-processing mechanisms in biological systems; the information processing units in the core/periphery structure are classified as core or peripheral units. The advantage of the core/periphery structure is that it helps reduce the costs for maintaining or changing services by distinguishing the service functions into core and peripheral functions. Unlike module-based design, a service functions network designed based on the core/periphery structure can accommodate different services while modifying only peripheral functions and reusing the core functions. In the long term, by keeping the core and periphery at appropriate sizes, this architecture can be expected to adapt to large increases in service functions and changes in commonly used functions. The method of maintaining the network based on the core/periphery structure can determine the structure of the network at the next stage based only on the topology of the service functions network at the current time. Therefore, it is expected to be able to continue to evolve at a stable and low cost, regardless of the inability to predict environmental changes.

Our previous works^11–13^ showed that network-oriented services designed based on a core/periphery structure can adapt to environmental changes with low development cost by reusing the core and recreating only the periphery. We numerically investigated the advantages of the core/periphery structure for accommodating information services, represented by chains of functions^11^. In addition, we focused on a shopping experience service using mixed reality and robots as a use case for realizing a service scenario based on^11^, and we implemented the service with an actual device and demonstrated that service design using a core/periphery structure is effective for robot operation when the number of device types on the user side and remote side increases^12,14^.

In this paper, we propose a method to evolve the entire network of service functions based on a core/periphery structure. Our goal is to maintain the service functions network that has high service ability while providing stability and low development cost. Service-providing ability is the ability to accommodate many new service chains and to accommodate services with short service chain lengths using only the service functions required. Shorter service chains lead to more responsive services. When all functions are connected sparsely, that is, when peripheral functions occupy the network, the development cost when adding new functions is small, but the chain length is long because it requires extra functions to accommodate service chains. When the service functions network is composed in a full mesh, that is, when core functions occupy the network, all service chains have a short length, but developers must add many interfaces to maintain a fully meshed network when adding new service functions. This makes the development cost significant and makes it impossible to maintain the services in the future.

Thus, we propose a method to maintain an appropriate scale of core and peripheral functions, with a trade-off between development costs and service-providing ability. We evaluated our method in terms of the service accommodation ratio and the service chain length to show that the service functions networks maintain a high service-providing ability when our method is applied. In addition, we evaluated the development costs required to evolve the service functions network. Our simulation results revealed that the service functions networks can continue to evolve with a low development cost.

**Figure 1:**
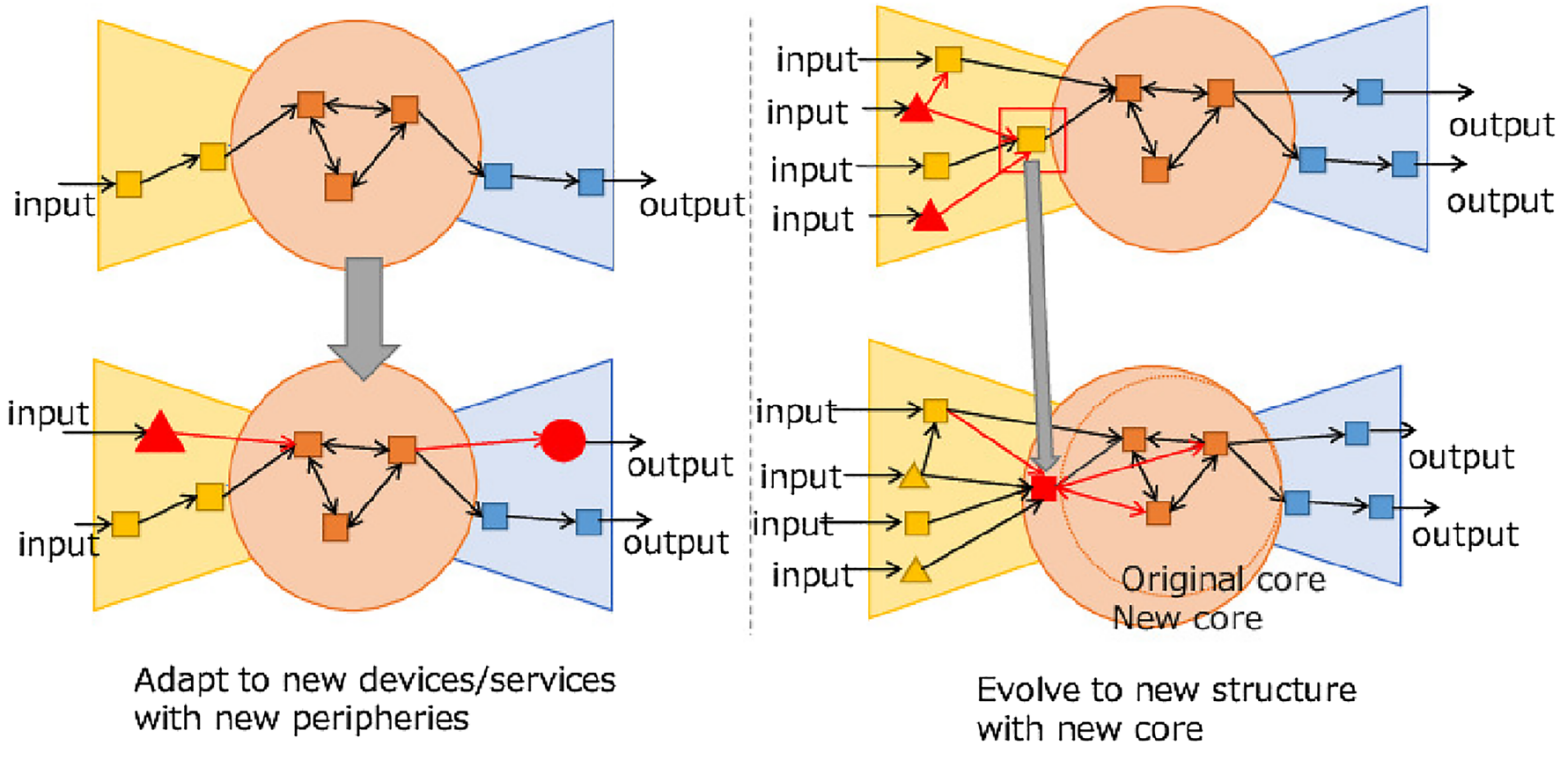
Overview of our proposed method.

Figure 1 shows our proposal based on a core/periphery structure. The left side represents a service functions network using a core/periphery structure that can adapt to new devices and services by changing only the periphery. The right side shows the method proposed in this paper, which evolves the entire service functions network by renewing the core.

Our contributions are as follows. (1) We propose a method to evolve a service functions network using a core/periphery structure. (2) We show that a service functions network can continue to evolve at low cost by maintaining the size and the density of a core and its peripheries.

The remainder of this paper is organized as follows. The section “Problem formulation of evolvable service functions network” describes our design problem and the problem formulation. The section “Density control of service functions network” describes our method to control the service functions networks. The “Evaluation” section describes the evaluation of our method. Finally, the “Conclusion” section provides some concluding remarks and areas of future study.

## Related work

### Algorithms with evolvability

Algorithms with evolvability include GAs and evolution strategies, which have been applied to a variety of problems^7^. In addition, Kashan et al.^8^ proposed modularly varying goals to make the GA adapt to varying environments. However, these algorithms are based on the presumption that the problem to be solved can be described as an optimization problem. The environmental changes that may occur in the future are too numerous to list, and it is difficult to mathematically represent future environmental changes as goals of optimization problems. We propose a method to evolve service functions networks so that they can adapt to unpredictable environmental changes.

### Accommodation of service chains

Many studies have been done on accommodating and deploying service chains for network function virtualization^15–18^. For example, Bian et al.^17^ proposed a distributed scheme that approximates the chain composition with a performance guarantee and adapts to resource failures in a timely manner. Cai et al.^18^ proposed an active service chain reconfiguration mechanism based on the computational load and resource demand. These studies only predict resource demand and other factors based on previous data, and do not continue to evolve the service functions network over the long term.

In the field of software development, as a design methodology to provide more services at less cost, module-based design has been widely introduced. In module-based design, modules connect to each other via interfaces, and developers can decouple and reuse them^19^. However, a disadvantage of module-based design is that the development becomes complex and the products become difficult to maintain. Albers et al.^20^ pointed out that the interdependencies among modules used in different products increase because changes to a module affects other products. As a result, the module’s development becomes complex, and it becomes difficult to maintain the module and products. Although Albers et al.^20^ focused on vehicle development, a module-based design of services also complicates the development. Modules are connected on an equal basis and have interdependencies. More importantly, modules, in our case service functions, are sometimes created by several different developers. Thus, it is difficult for developers to observe the scope of the effects of changes when they modify their modules. This will lead to maintenance difficulties and modification of other services and/or modules. Thus, the entire service functions network developed by different developers needs to be adaptable to accommodate many new services.

## Problem formulation of evolvable service functions network

In this paper, we assume that there are many service functions created by software developers, and that some of the service functions are connected to other functions through interfaces such as APIs. We describe our system model and define its evaluation metrics in this section.

### System model

#### Service functions network and service chain

**Figure 2:**
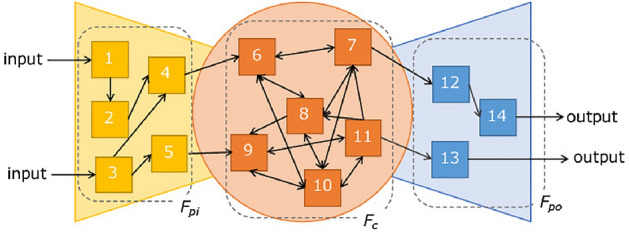
Example of service functions network $G_t$.

**Figure 3:**
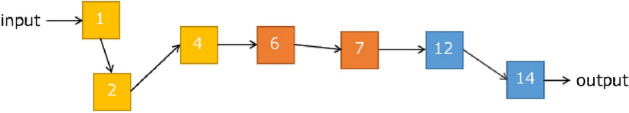
Example of $$G'_t$$.

We consider a network consisting of service functions and interfaces that connect them. The network of service functions at time *t* is represented by a directed graph $G_t(\{F_{pit}, F_{ct}, F_{pot}\}, L_t)$ consisting of a set of input-side peripheral functions $F_{pit}$, a set of output-side peripheral functions $F_{pot}$, a set of core functions $F_{ct}$, and a set of links $L_t$. Each node in the network represents a service function, which works independently of other functions. Links $L_t$ represent the set of links among the service functions. When an interface is available from service function *i* to service function *j* and *i* can call *j*, a link (*i*, *j*) exists. The service providers attempt to connect the service functions they use based on their input data and the output data they want to acquire. The directed graph connecting those service functions from the input side to the output side is a service chain. We call a service chain $sc_t$ using existing interfaces a known service chain. For instance, given a service function network $G_t$ in Fig. 2, the chain shown in Fig. 3 is a known chain and a subgraph of $G_t$. A known service chain is represented by a directed graph $G'_t(\{F'_{pit}, F'_{pot}, F'_{ct}\}, L'_t)$. $G'_t$ is a subgraph of $G_t$ because $F'_{pit}, F'_{pot}, F'_{ct}$, and $L'_t$ are subsets of $F_{pit}, F_{pot}, F_{ct}$, and $L_t$ respectively. Here, we assume that the functions in $F_{pi}$ are not used after the functions in $F_{po}$ and the functions in $F_{c}$ and $F_{pi}$ are not used after the functions in $F_{po}$.

#### Service accommodation

**Figure 4:**
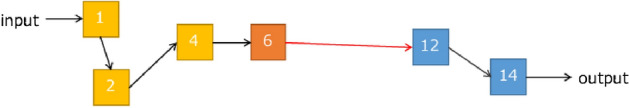
Example of a service chain that is accommodated by using other service functions and interfaces.

**Figure 5:**
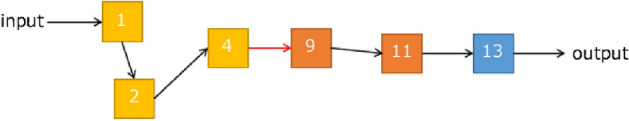
Example of a service chain that is not accommodated.

This section describes the accommodation of service chains. In this paper, we assume that a service chain can be accommodated when there is a combination of interfaces that allows the service functions included in a service chain to be used in the given order. All of $G'_t$ and some service chains that are not subgraphs of $G_t$ are accommodated. For example, the chain in Fig. 4 is unknown, but it can be accommodated because service function 7 can be used between functions 6 and 12. However, in this case, service functions and communications that are not originally required are used. The link (6, 12) provides a shorter chain and more efficient service. We call the minimum number of interfaces required to accommodate a given service chain $sc_t$ with a chain length of $l_{sct}$. Service chains that have a combination of unreachable service functions, as shown in Fig. 5, are not accommodated.

### Definitions of metrics

#### Development cost

To increase the probability of accommodation of the service chain and to provide services with short chain lengths, additional interfaces between service functions are required. Based on the information available at time *t*, we determine interfaces $L_{t+1}$ and add a link. We define the development cost *C* required to accommodate a service chain by the number of links to be added.

1 $$\begin{aligned} C= |L_{t+1} \setminus L_t|. \end{aligned}$$

#### Service accommodation ratio

Let $ac_{sc}=1$ when the service chain *sc* can be accommodated in $G_t$ and $ac_{sc}=0$ otherwise. For the set of service chains *SC* arising at time *t*, the accommodation ratio *AC* is

2 $$\begin{aligned} AC = \frac{\sum _{sc \in SC}ac_{sc}}{|SC|}. \end{aligned}$$

Our goal is to maximize $\alpha AC + \beta C$, which represents the service accommodation ratio for the same *C* when *t* is increased. $\alpha > 0$ and $\beta < 0$ are parameters for weight.

We list our symbols in Table 1.

**Table 1:** Symbol definitions.

Name	Description
$F_{pi}$	Set of input-side peripheral functions
$F_{po}$	Set of output-side peripheral functions
$F_{p}$	$F_{in} \cup F_{out}$
$F_{c}$	Set of core functions
*coresize*	Ratio of cores to the total network
*L*	Set of links
$d_{\{pi, po, c\}}$	Density within the function block
$d_{(c, \{pi, po\})}$	Density between the function blocks
*C*	Development cost
*sc*	A service chain
$l_{sc}$	Chain length

## Density control of service functions network

When we consider the realization of an evolvable service functions network, it is difficult to solve the problem as an optimization problem because it is difficult to express unknown future service requirements in mathematical form. Therefore, we enable service functions networks to evolve by maintaining a core/periphery structure of appropriate size and density. We refer to this as “density control”.

This section describes our method of determining interfaces $L_{t+1}$.

### Approach based on density control

In Gu et al.^21^, for two given blocks in the human brain, when (connection strength within block *m*) > (connection strength between blocks *mandn*) > (connection strength within block *n*), block *m* is a core block and *n* is a peripheral block. Gu et al.^21^ shows that in terms of the organization of brain functions, as the brain develops, modules become more separate, the number of core-periphery pairs increases, and the strength of the connections within blocks and the strength of the connections between blocks are negatively correlated. That is, the more separated they are, the more they indicate a state of well-developed function. In the service function relationship, a negative correlation between the density between blocks and the density within a block facilitates development of the function of each block because of its small dependencies on other blocks.

It is important that the density between the blocks is between the density of the core block and the density of the peripheral blocks. Gu et al.^21^ explains that, when the density between blocks is greatest, they are not separated and are a monolithic structure, and when the density between blocks is smallest, there is no interrelationship between them. That is, the density between the core block and the peripheral block is required to be sufficiently smaller than the density within the core block to facilitate development, but sufficiently greater than the density within the peripheral blocks to accommodate service chains.

In addition, this approach enables to distinguish core blocks from periphery blocks and determine $L_{t+1}$ from only the network topology information regardless of the content of the service function or the frequency of use. Determining the service functions network by distinguishing the roles of the core and periphery based on the frequency of use of the service and the content of the service function is conceivable, but it is difficult to obtain the information for all service functions and predict future information.

In a service functions network, the role of the service function can change to accommodate unknown service chains over time. Therefore, we control the service functions network so that some of the peripheral functions are newly regarded as core functions while keeping the inequalities.

### Service functions network based on a core/periphery structure

The connection between function blocks is represented by an adjacency matrix *A* of blocks *m* and *n*. $A_{ij}=1$ when the interfaces $i\in m$ to $j\in n$ are available, and $A_{ij}=0$ when they are not. When $m=n$, that is, within $F_{pi}$, $F_{po}$, and $F_c$, we define $d_m$, the density within function block *m*, as

3 $$\begin{aligned} d_{m}= \frac{\sum _{i\in m, j\in m, i\ne j}A_{ij}}{|m|\cdot |m| - 1}. \end{aligned}$$

We define $d_{m, n}$, the density between blocks, as

4 $$\begin{aligned} d_{m, n}= \frac{\sum _{i\in m, j\in n}A_{ij}}{|m|\cdot |n|}. \end{aligned}$$

Hereafter, we refer to the density within $F_{pi}, F_{po},and\ F_{c}$ as $d_{pi}, d_{po}, and\ d_c$, respectively, and the density between a core block and peripheral block as $d_{c, pi}\ and\ d_{c, po}$, respectively.

**Figure 6:**
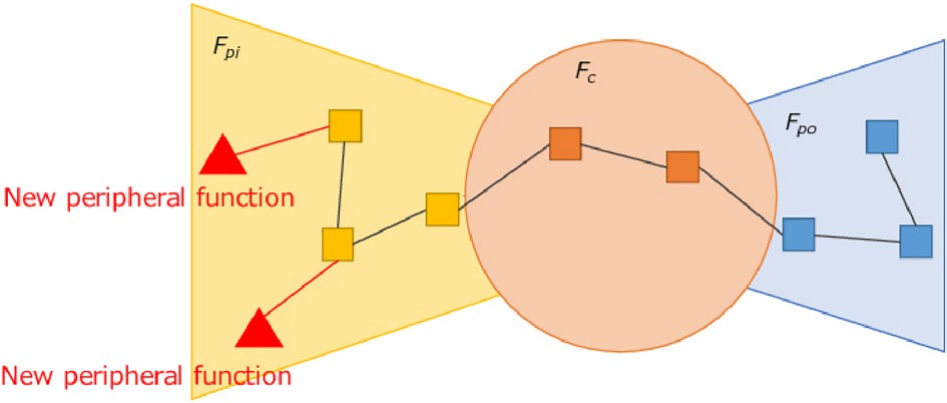
Example of service functions before application of the method.

**Figure 7:**
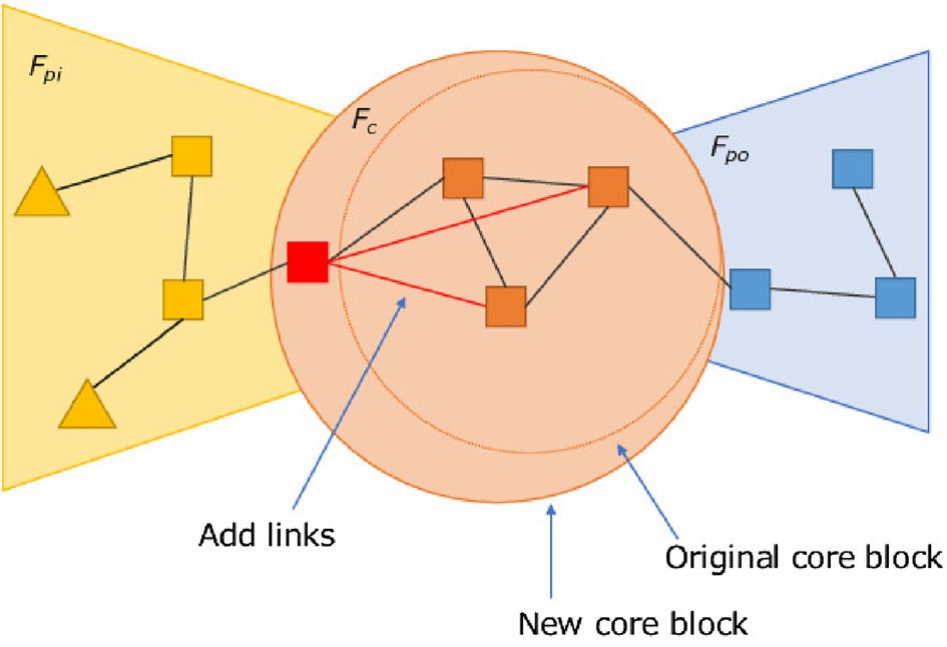
Example of service functions after adding a core function.

**Figure 8:**
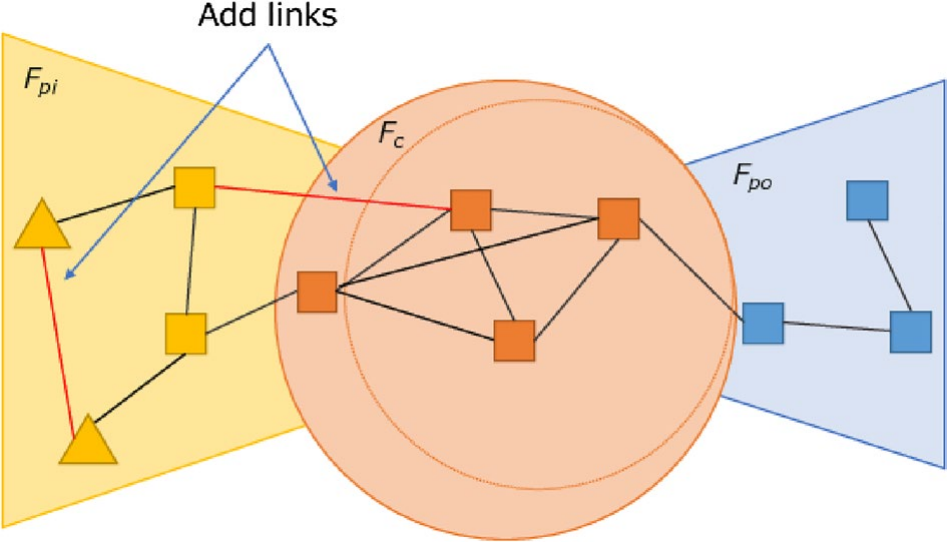
Example of service functions after adding links between peripheral functions.

### Adding links to accommodate unknown service chains

We focus on the service function $fp \in F_{p}$, where *fp* is connected to one or more of the service functions in $F_{c}$. Let $L_{fp, c}$ be the set of links connecting *fp* and $F_{c}$, and $L_{fp, p}$ be the set of links connecting *fp* and the block where *fp* belongs.

$G_{t+1}$ is determined by Algorithm 1. In our simulations, we control the density in the following order, but we can get the same results if the order is switched. Figure 6 shows an example of the service functions network before the method is applied, as the peripheral functions increase on the input side. In lines 1–5, we control $d_c$. We select two functions at random in $F_c$ and add links between them. Our method randomly selects which nodes to link. The nodes to which links are added can be determined based on information such as the frequency of use or degree. Because service chains that will arise in the future are difficult to predict, adding links randomly leads to accommodating a variety of service chains. To make the density of core blocks sufficiently greater than the density between blocks, we add links until the following conditions are met: $d_{c}-d_{c, pi} > th_{c,cp}$, $d_{c}-d_{c,po} > th_{c,cp}$, and $d_c \ge dmin_c$. The core functions require a sufficiently higher density than the density between the core and peripheral blocks, because the availability of a sufficiently densely connected function block provides a shorter chain length, that is, a more efficient service. Figure 7 shows an example of the service functions network when this process is complete.

In lines 6–11, we control the core size. We randomly select $fp \in F_{p}$ and nodes in $F_c$ and add links between them so that *fp* can be considered as $fp \in F_{c}$. $F_c$ including *fp* becomes the new core block. The system transitions to the next state when at least one of following conditions is met: $coresize \ge coresize_{max}$ or ($d_{c} - d_{c, cpi} > th_{c,cp}$ and $d_{c} - d_{c, cpo} > th_{c,cp}$). The size of the core is required to be within a certain range, because maintaining a large core requires a large number of additional links, which increases the development cost, and too small a core cannot maintain sufficient service-providing ability (accommodation ratio and chain length).


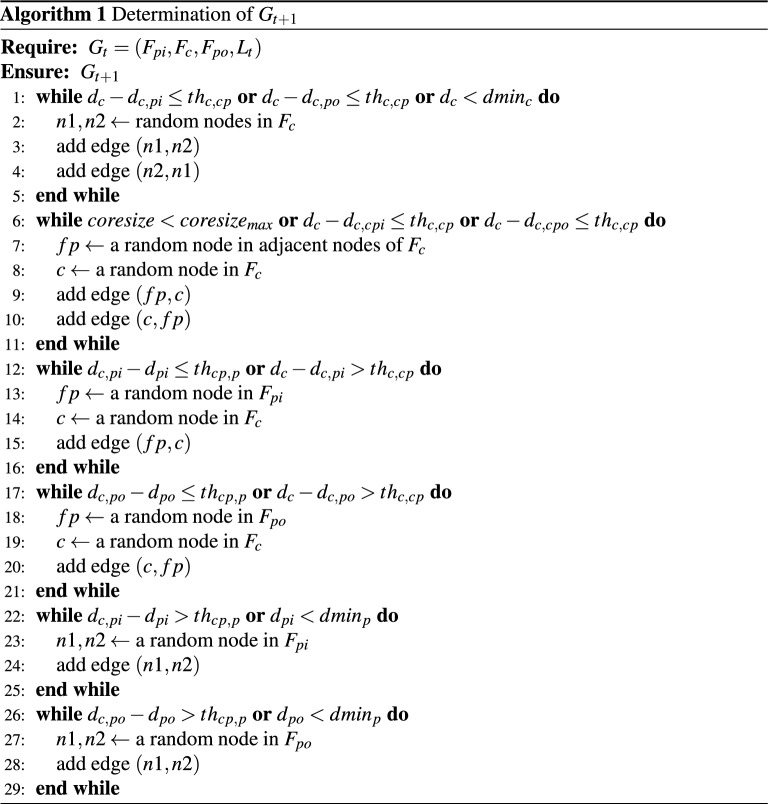


In lines 12–21, we control $d_{c, pi}$ and $d_{c, po}$. We randomly select nodes in $F_{p}$ and nodes in new $F_c$ and add the links between them. To make the density between blocks sufficiently greater than the density within peripheral blocks, we add links until the following conditions are met: $d_{c, pi}-d_{pi} > th_{cp,p}$, $d_{c}-d_{c,pi} \le th_{c,cp}$, $d_{c, po}-d_{po} > th_{cp,p}$, and $d_{c}-d_{c,po} \le th_{c,cp}$. We set the second and fourth conditions to avoid adding an unlimited number of links.

In lines 22–29, we control $d_{pi}$ and $d_{po}$. We randomly select peripheral nodes in the same block and add links between them. To make the density within peripheral blocks sufficiently great to become function blocks, we add links until the following conditions are met: $d_{c, pi}-d_{pi} \le th_{cp,p}$, $d_{pi} \ge dmin_p$, $d_{c, po}-d_{po} \le th_{cp,p}$, and $d_{po} \ge dmin_p$. We set the second and fourth conditions to avoid adding an unlimited number of links. Figure 8 shows an example of the service functions network when this process is complete.

## Evaluation

### Comparable methods

We evaluated the effectiveness of our density control method by comparing it with three other methods. The first one is the “shortest-path accommodation” method. This method adds links to connect all nodes of each service chain so that each service chain at *t* is a subgraph of $G_t$. The chain length is minimized for the service chains accommodated, but the development cost is high because links must be added for all the different service chains. Thus, we compare service accommodation ratios when the same development cost is used for density control. The second method is “low-cost accommodation”. Density control requires a development cost to add links between service functions, regardless of whether a service chain occurs because it maintains the size and density of the core and peripheral functions. Low-cost accommodation enables service chains occurring at *t* with as low a development cost as possible. Specifically, we calculate how much the accommodation ratio is increased by each link between all nodes, and add the link with the largest value. We repeat it until no more links are available to increase the accommodation ratio. The third method is “random”, in which nodes are selected randomly and links are added between them with the same development cost as that of density control. Note that the links that the methods except for shortest-path accommodation can add are those from $F_{pi}$ to $F_c$, $F_{pi}$ to $F_{po}$, or $F_{c}$ to $F_{po}$.

Our simulation program executes the algorithm in the following order at each step *t*. First, we give the initial state of the service functions network at *t*. The network consists of a core block $F_c$, an input-side peripheral block $F_{pi}$, and an output-side peripheral block $F_{po}$. Second, environmental change at *t* occurs. We add peripheral functions with probability *p*. Specifically, we generate a random number *r*. When $\frac{2}{p}<r\le p$, a function is randomly selected from $F_{pi}$, and otherwise a function is randomly selected from $F_{po}$. Then, the new function is connected to the selected node as a leaf. We add up to three additional peripheral functions during each step. Third, each method determines $G_{t+1}$, and their development cost and accommodation ratio *AC* are calculated. $G_{t} \leftarrow G_{t+1}$ for each method.

### Evaluation metrics

We evaluated the methods with the following three metrics.

First, we calculated the accommodation ratio of the service chains to evaluate the ability of our method to accommodate new service chains. We assumed that the random and shortest-path accommodation methods can use the same development cost as our density control method at each step.

We created 30 service chains for each step in the following way. First, we selected one input node and one output node at random. The nodes in $F_p$ that are leaves are candidates for input and output nodes of service chains, respectively. Second, we randomly selected nodes to generate the service chain from all nodes other than the input and output nodes. The number of nodes at step *t* are $N_t$. The number of nodes selected here is set to be between 1 and $\frac{N_i}{3}$. Note that they are connected in the order of the nodes in $F_{pi}$, nodes in $F_c$, and nodes in $F_{po}$. The chain connecting the input nodes, the selected nodes, and the output nodes is a service chain. Here, 15 of the service chains are selected based on the core/periphery classification at $t=0$, and the remaining 15 are selected based on the core/periphery classification when density control is applied. This is to focus on new service chains that use core functions that were not previously used as core functions.

Second, we used the development cost. The low-cost accommodation method tries to accommodate the service chains with as little cost as possible, so its development cost is less than that of density control. To show that our method can change the structure of the service functions network at a sufficiently low development cost, we evaluated the development cost. We calculated the development cost of each method to achieve its objectives at each step to evaluate how much more development cost the density control and shortest-path accommodation methods require compared to the low-cost accommodation method to accommodate the same service chains. We calculated the development cost of accommodating all service chains generated at each *t* in the shortest distance with unlimited development cost available for the shortest-path accommodation method. The development costs of other methods are same as explained in the section on “Problem formulation of evolvable service functions network”.

Third, we used the chain length. In the low-cost accommodation method, *AC* is expected to be higher, but it has the disadvantage of requiring extra paths to be taken to accommodate the service chain. Longer chains mean the use of service functions and communication paths that are not required to provide the service, which leads to degradation of the responsiveness of the service. Therefore, we calculated the chain length $l_{sc}$ with our density control method and the length with the low-cost accommodation method. For a service chain *sc* consisting of $N_{sc}$ functions, the minimum value is $N_{sc}-1$ when the given service chain is a subgraph of $G_t$. In this simulation, we calculated $\frac{l_{sc}}{N_{sc}-1}$, which represents how many times longer the chain length $l_{sc}$ is compared to $N_{sc} -1$, the minimum chain length, as an indicator of the service responsiveness.

### Simulation results

**Figure 9:**
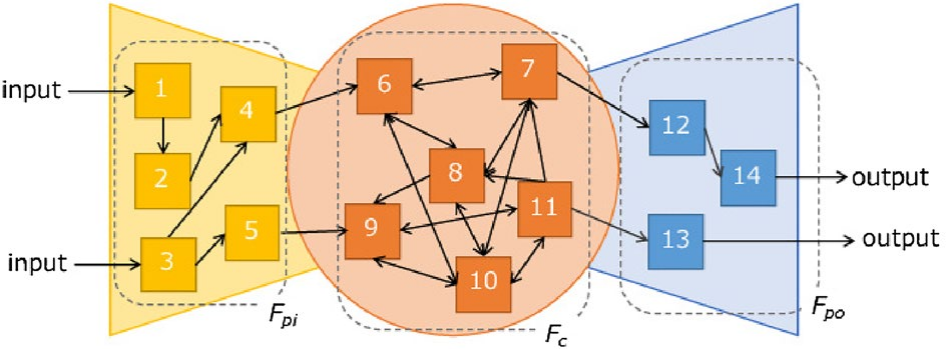
Service function network at $t=0$.

We used the network represented in Fig. 9 and parameters shown in Table 2. We executed the methods until $t=40$ and repeated this 100 times.

**Table 2:** Parameter settings for evaluation.

Parameter	Description	Setting
$coresize_{min}$	Minimum ratio of cores to the total network	0.3
$coresize_{max}$	Max ratio of cores to the total network	0.4
*p*	Probability of new peripheral functions to be created	0.4
$th_{c,cp}$	Threshold of $d_c-d_{c, pi(or\ po)}$	0.3
$th_{cp, p}$	Threshold of $d_{c, pi( or\ po)}-d_{pi (or\ po)}$	0.2
$dmin_{cp}$	Minimum value of $d_{c, pi (or\ po)}$	0.3
$dmin_{p}$	Minimum value of $d_{pi (or\ po)}$	0.2
$dmin_{c}$	Minimum value of $d_{c}$	0.7
$dmax_{c}$	Maximum value of $d_{c}$	0.8

#### Service accommodation

**Figure 10:**
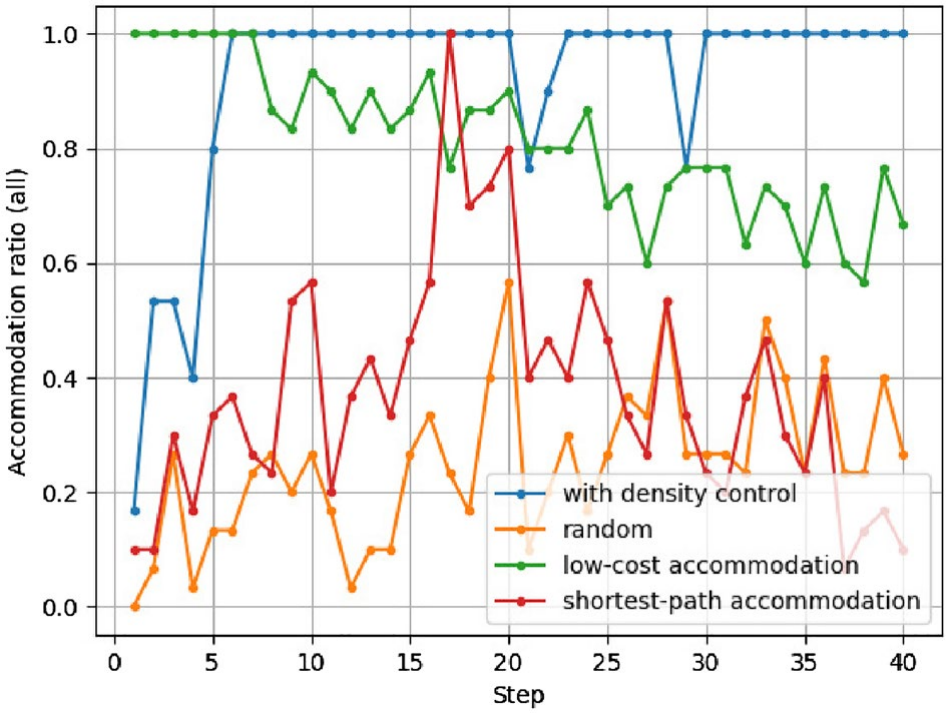
Example of accommodation ratios for all service chains.

**Figure 11:**
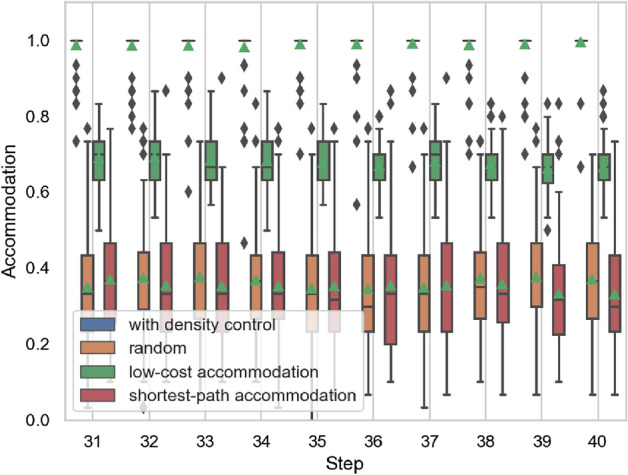
Accommodation ratios for all service chains.

The results of the accommodation ratio calculations are shown in Figs. 10 and 11. The accommodation ratio for an example of the evolution of all paths for each service functions network is shown in Fig. 10. Figure 11 shows the accommodation ratio for 100 executions of each method, that is, the accommodation ratio for following 100 evolutionary paths. We excerpt the 30th and later steps, when the effect of the initial network state has diminished. The horizontal axis represents the evolutionary steps, and the vertical axis represents *AC*. Note that the random and shortest-path accommodation methods can use the same development cost as our density control method at each step.

As the steps are executed, the accommodation ratio approaches nearly 1 when density control is applied, but that with the low-cost accommodation method decreases. This is because there are fewer nodes that are bi-directionally linked (i.e., less functions that are used mutually), and the network cannot accommodate the service chains that do not use new core functions. In the density control method, the number of combinations of functions that can be used mutually is increased by densely connecting peripheral functions as new core functions. As a result, it is now possible to accommodate service chains that use core functions that were not previously part of the core.

The accommodation ratio is not stable when the nodes in the service chain arriving at each step are connected using the same development cost as our density control method. As the size of the network increases over the course of the evolution, the length of service chains increases. Because the shortest-path accommodation method incurs a higher development cost to accommodate longer service chains, the accommodation ratio is lower when more long service chains arise.

When density control method is applied, the accommodation ratio is close to 1.0 for almost all evolutionary paths. When shortest-path accommodation and random methods are applied, the variance in accommodation ratios is large. Each method takes same cost for additional APIs (links) between service functions to accommodate emerged service chains. Shortest-path accommodation method tries to minimize the chain length at the step without considering possible future use of APIs. Our results on accommodation ratios shows that, in shortest-path accommodation method, few links added at previous steps are reused when accommodating a new service chain. Random also has a large variation in service accommodation ratio. Density control also uses randomness in some processes. However, the fact that the accommodation ratio does not increase with the random method, which adds links completely at random. This result indicates that it is effective to maintain the relationship between core and peripheries scale based on a core/periphery structure.

#### Development cost

**Figure 12:**
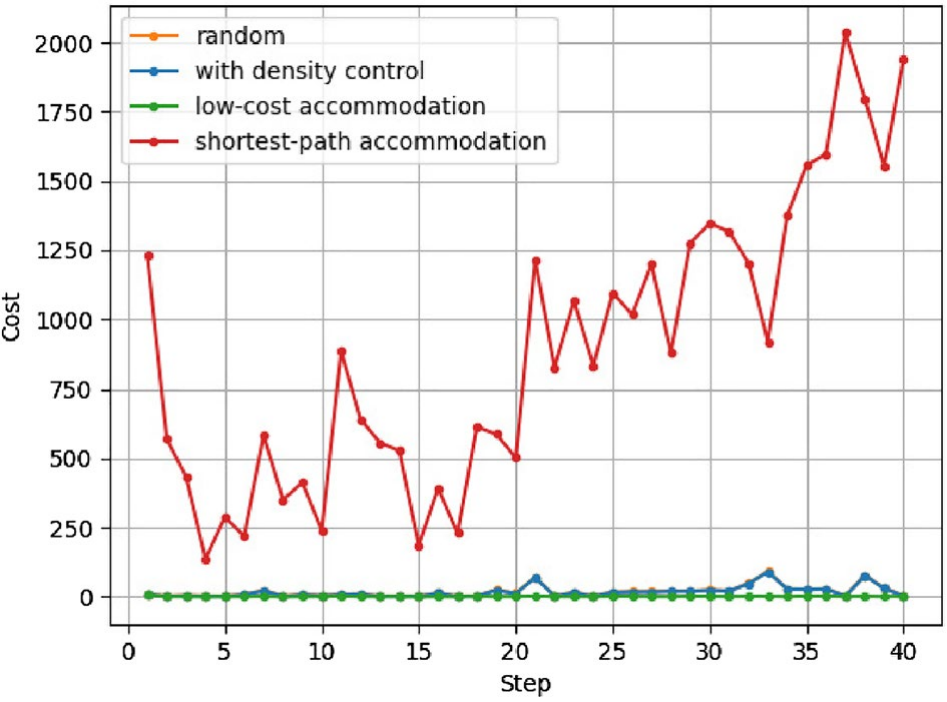
Example of development cost.

**Figure 13:**
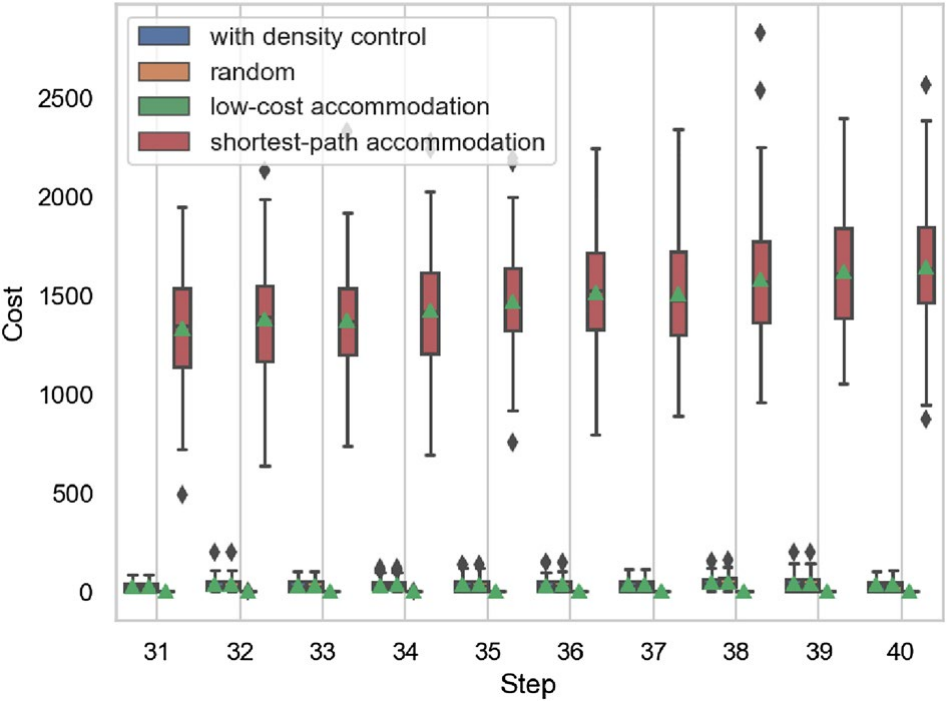
Development cost.

The results of calculating the development cost at each step are shown in Figs. 12 and 13. The horizontal axis represents evolutionary steps, and the vertical axis represents the development cost of each step.

The density control method adds up to about 10 times more links than the low-cost accommodation method with our settings. However, low-cost accommodation calculates accommodation ratios for all possible links. The larger the service functions network, the more difficult it becomes to find the optimal link to add from the vast possible combinations of nodes.

The cost for the shortest-path accommodation method shown in Fig. 12 is the development cost required to accommodate all service chains, including those not accommodated, as shown in Figs. 10 and 11. The development cost for the shortest-path accommodation method increases significantly because the links added at *t* are rarely reused in the future. The development cost for low-cost accommodation and shortest-path accommodation are highly dependent on the parameter settings, such as the length and number of service chains arriving at each *t* and it is difficult to predict what service chains will occur in the future. However, the development cost for density control is not dependent on the content of the service chain because it is based only on network topology.

#### Chain length to accommodate services

**Figure 14:**
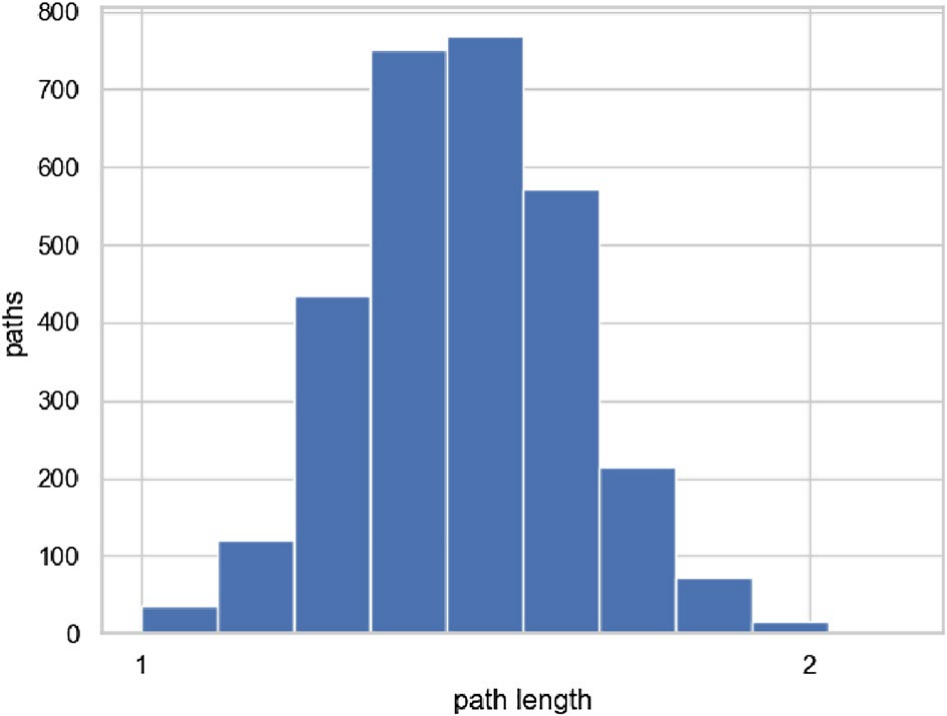
Chain length (density control).

**Figure 15:**
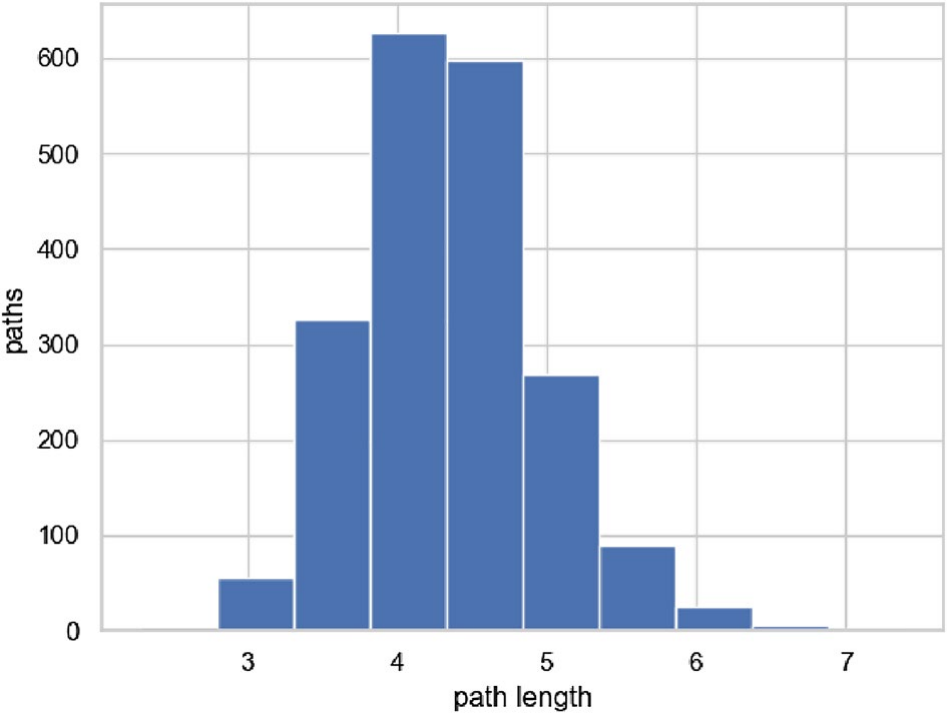
Chain length (low-cost accommodation).

The lengths of the chains with density control and low-cost accommodation are shown in Figs. 14 and 15, respectively. The density control method requires only 1–2 times the shortest length of the service chains because we control the density between $F_c$ and $F_{p}$. The low-cost accommodation method requires about 3–5 times the shortest length of the service chains. It adds links between peripheral functions because it determines the links based on the accommodation ratio. As a result, the peripheral functions become closer to being connected in a ring-like shape. If the low-cost accommodation method is modified to have a shorter chain length, it performs the same process as the shortest-path method, and the development cost becomes higher as shown in Figs. 12 and 13.

### Evolvability of service design

Our simulation results revealed that our proposed method enables the service functions network to evolve to accommodate more new service chains. The density control method achieves stable and high service chain accommodation ratios in multiple evolution paths. In addition, the development cost used to apply density control is independent of the number or length of future service chains. This provides an advantage for changing the structure of the service functions network in the future for a long period of time, because other methods require additional development cost to accommodate new services depending on the number or length of service chains, and it is difficult to predict what service chains will arise in the future. We expect that this advantage becomes more significant as the size of the service functions network and service chains become larger.

We set the development frequency of new peripheral functions as about one month per step. That is, our simulation results show the service accommodation ratio and development cost for about 40 months. Applying our method to the service functions network enables the network to evolve at lower cost, which facilitates the development of services more quickly. Then, because each evolutionary step is shorter, many steps can be taken in the same 40 months, as in our simulation. As more steps are taken, the difference from low-cost accommodation in terms of service accommodation ratio increases, and the difference from shortest-path accommodation in terms of development cost increases. Therefore, the advantage of our method is greater.

The time complexity of our proposed method is $O(N_t)$, where $N_t$ is the number of nodes at time *t*, because all loops used in density control are single-loop. Those of comparison method shortest-path accommodation is $O(N_{sc})$ where $N_{sc}$ is the number of nodes of service chains. The complexity of low-cost accommodation is $O(N^2)$ because it verifies all possible links. The space complexity of our proposed method is $O(N_t)$, because it only requires the service functions networks for the input and constant number of variables to store the nodes to add links. Those of the comparable methods are $O(N_t)$ as well. Therefore, the time/space complexity of our proposed method is not as high as that of other methods.

## Conclusion

To accommodate large numbers of services at low cost, the service design needs to be adaptable to user requirements and environmental changes. Our previous works have shown that network-oriented services designed based on a core/periphery structure can adapt to environmental changes with a low development cost by implementing a specific network-oriented service. However, in practice, service providers combine various service functions developed by several developers via interfaces such as APIs, and provide the service to users. Therefore, the entire network of service functions is required to adapt to environmental changes that are difficult to predict. In this paper, we proposed a method to evolve the entire service functions network. Our method evolves the service function network with low development cost by keeping the core and peripheral functions at the appropriate scale. We evaluated our method in terms of the service accommodation ratio and the service chain length to show that the service function networks maintain a high service-providing ability by applying our method. In addition, we evaluated the development cost required to evolve the service functions network. Our simulation results revealed that our proposed method enables the service functions network to continue evolving and accommodate more new service chains with shorter chain lengths. Future work includes addressing the problem of where to place service functions and providing a method to adapt the service functions network to the unavailability of some service functions.

## Data Availability

The data in this study are available from the corresponding author on reasonable request.
